# Confocal endoscopy-assisted diagnosis of duodenal follicular lymphoma: a rare case report

**DOI:** 10.1055/a-2590-8569

**Published:** 2025-06-13

**Authors:** Lilan Fan, Xing Huang, Jun Luo, Lijing Yang, Qiu Zhao, Jing Liu

**Affiliations:** 1 Department of Gastroenterology, Zhongnan Hospital of Wuhan University, Hubei Clinical Center and Key Lab of Intestinal and Colorectal Diseases, Wuchang District, Wuhan City, Hubei Province, China; 2 Center for Pathology and Molecular Diagnostics, Wuhan University, Wuhan, China; 3 89674 Department of Pathology, Zhongnan Hospital of Wuhan University, Wuhan, Hubei, China; 4 89674 Department of Radiation and Medical Oncology, Zhongnan Hospital of Wuhan University, Wuhan, Hubei, China

Duodenal follicular lymphoma (D-FL), a rare gastrointestinal lymphoma, poses diagnostic challenges due to nonspecific symptoms 1 2 . This case highlights the utility of confocal laser endomicroscopy (CLE) in early detection and management.

A 59-year-old woman with bronchial asthma presented with epigastric pain and bloating. Gastroscopy revealed mucosal thickening and erosions at the duodenal bulb-descending junction ( Fig. 1 **a** ). CLE identified disrupted glandular architecture, dilated lymphatic vessels, and fluorescein leakage, prompting targeted biopsies ( Fig. 1 **b** , Video 1 ). Histopathology confirmed neoplastic follicles ( Fig. 2 ), while immunohistochemistry (CD20+, CD10+, Bcl-6+, BCL2+, and Ki-67+) and IG gene rearrangement confirmed follicular lymphoma. PET/CT ( Fig. 3 **a** ) and abdominal CT ( Fig. 3 **b** ) localized disease to the duodenum without extraintestinal involvement (stage II).

**Fig. 1:**
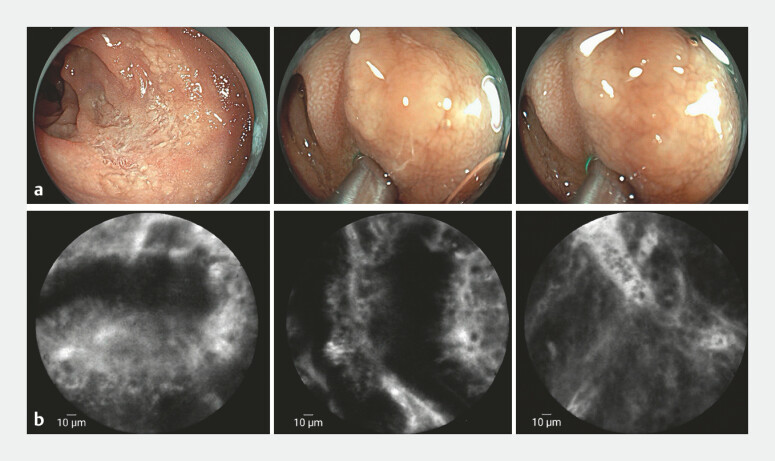
Endoscopic image. **a** Gastroscopy showing scattered white irregular erosive lesions and mucosal thickening at the junction of the duodenal bulb and descending duodenum. **b** CLE revealing irregular glandular structures, focal loss of glandular architecture, thickening and dilation of lymphatic vessels, disorganized tissue arrangement, and increased fluorescein sodium leakage. Abbreviation: CLE, confocal laser endomicroscopy.

Confocal laser endomicroscopy in the application of duodenal follicular lymphoma.Video 1

**Fig. 2:**
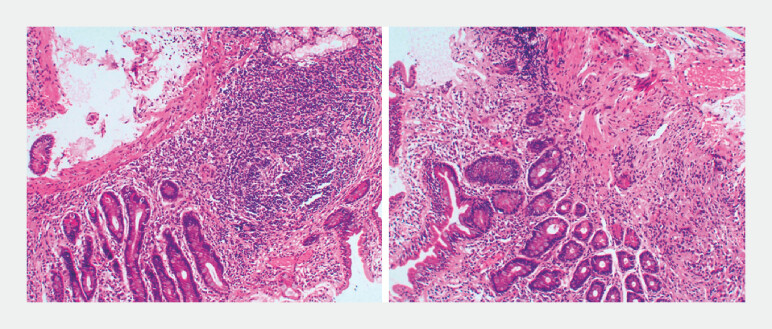
Histopathological examination. Histopathological examination demonstrating neoplastic follicles, consistent with FL.Abbreviation: FL, follicular lymphoma.

**Fig. 3:**
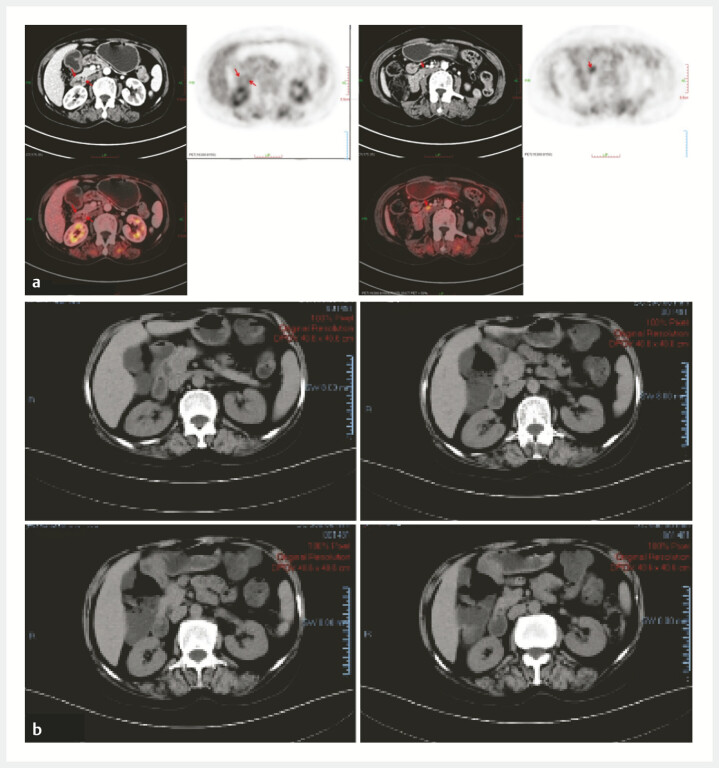
Imaging examination. **a** PET/CT scan demonstrating uneven thickening of the intestinal walls in the descending and horizontal sections of the duodenum, along with multiple areas of abnormal uptake within the abdominal cavity. **b** Abdominal enhanced CT scan confirming the absence of extraintestinal involvement, with no evidence of abnormal lesions or extension beyond the intestinal walls.

Treatment began with Rituximab-CVP (cyclophosphamide, vincristine, and prednisone) for four cycles, achieving remission. Due to asthma-related concerns, therapy was switched to Rituximab-Lenalidomide, which was well-tolerated. Regular follow-up was advised to monitor progression.

CLE’s real-time, high-resolution imaging enabled early detection of microarchitectural abnormalities, guiding precise biopsies and minimizing invasive sampling. Multimodal imaging (PET/CT, CT) confirmed localized disease, underscoring its role in staging. This case illustrates the synergy of advanced endoscopy, histopathology, and imaging in diagnosing D-FL. Personalized treatment, balancing efficacy and comorbidities, optimized outcomes without compromising safety.

In conclusion, CLE enhances early diagnosis of D-FL by visualizing microscopic changes, complementing traditional methods. Combined with tailored therapies and rigorous follow-up, it improves diagnostic accuracy and long-term management in rare gastrointestinal malignancies.

Endoscopy_UCTN_Code_CCL_1AB_2AZ_3AB

## Notes

Endoscopy E-Videos https://eref.thieme.de/e-videos
*E-Videos* is an open access online section of the journal *Endoscopy* , reporting on interesting cases and new techniques in gastroenterological endoscopy. All papers include a high-quality video and are published with a Creative Commons CC-BY license. Endoscopy E-Videos qualify for HINARI discounts and waivers and eligibility is automatically checked during the submission process. We grant 100% waivers to articles whose corresponding authors are based in Group A countries and 50% waivers to those who are based in Group B countries as classified by Research4Life (see: https://www.research4life.org/access/eligibility/ ). This section has its own submission website at https://mc.manuscriptcentral.com/e-videos .
